# Prevalence and genotype distribution of human papillomavirus in 961,029 screening tests in southeastern China (Zhejiang Province) between 2011 and 2015

**DOI:** 10.1038/s41598-017-13299-y

**Published:** 2017-11-01

**Authors:** Xiao Chen, Haiou Xu, Wanwan Xu, Wenjie Zeng, Jinwei Liu, Qing Wu, Xiaofeng Zhao, Tang Jiang

**Affiliations:** 1 State Key Laboratory for Diagnosis and Treatment of Infectious Diseases, Collaborative Innovation Center for Diagnosis and Treatment of Infectious Diseases, Key Laboratory of Clinical In Vitro Diagnostic Techniques of Zhejiang Province, the First Affiliated Hospital, College of Medicine, Zhejiang University, Hangzhou, Zhejiang 310003 China; 2 Department of Gynecology, Zhejiang Provincial People’s Hospital, Hangzhou, Zhejiang 310014 China; 3 Bengbu Medical College, Department of Gynecology, Zhejiang Provincial People’s Hospital, Hangzhou, Zhejiang 310014 China; 4 Key Laboratory of Tumor Molecular Diagnosis and Individualized Medicine of Zhejiang Province, Departments of Gynecology, Zhejiang Provincial People’s Hospital, Hangzhou, Zhejiang 310014 China; 5 Hangzhou Medical Laboratory of Dian Diagnostics, Hangzhou, Zhejiang 310023 China

## Abstract

Human papillomavirus infection plays a key role in the development of cervical cancer. To establish a foundation for HPV-based screening and vaccination programs, we investigated the HPV prevalence and genotypic distributions in Chinese women from Zhejiang Province. Between 2011 and 2015, a total of 961,029 samples from 2021 clinical hospitals were tested HPV genotype by a PCR-based hybridization gene chip assay, and 443,890 samples were evaluated cervical cytology by liquid-based cytology analysis. Our results showed that the positive rate for HPV was 20.54%, which ranged from 28.72% to 17.81% and varied by year of recruitment. Age-specific prevalence showed a “two-peak” pattern, with the ≤20-year-old group presenting the highest HPV infection rate, followed by 61–70-year-old group. Overall, the most prevalent genotypes were HPV16, 52 and 58. Additionally, the odds ratios for the prevalence of the HR-HPV, LR-HPV and HPV-negative groups with abnormal cytology were 12.56, 3.21 and 0.06, respectively. Among genotypes, HPV 16 has been found to have the highest OR, followed by HPV58, 18, 52. Here, we present data regarding the prevalence and type distribution of HPV infection, which can serve as valuable reference to guide nationwide cervical cancer screening and HPV vaccination programs.

## Introduction

Cervical cancer is the second most commonly diagnosed cancer and the third leading cause of cancer death among females in developing countries^1^. In China, cervical cancer is also the second most common gynecologic malignancy, and the incidence and mortality rates of cervical cancer in young women are increasing^2^. Cervical cancer is usually caused by human papillomavirus (HPV) infection. More than 200 HPV genotypes have been described, and approximately 40 are responsible for genital infection^3^. Although most HPV infections are transient and may resolve spontaneously, persistent infections caused by a subset of HPV genotypes are lead to the development of cervical cancer and its precursors^4^. On the base of the association with premalignant and malignant lesions, genital HPV is classified as low or high risk, respectively. The low-risk types, such as HPV6, 11, 40, 42, 43 and 44, are associated with genital warts. The high-risk types include 16, 18, 31, 33, 35, 39, 45, 51, 52, 56, 58, 59, 68, 73 and 82, and they contribute to 96.6% of invasive cervical cancer diagnosed worldwide^5^. HPV screening, especially for high-risk HPV (HR-HPV), may reduce the risk of cervical cancer^6^. The distribution and prevalence of HPV has been reported to vary by geographic region, and even among different areas in the same country^3,7^. Geographical differences in HPV distribution may affect the effectiveness of the HPV vaccine in different populations and different age groups^8^. Regional data on the prevalence and type distribution of HPV are essential for estimating the impact of vaccines on cervical cancer. Although a series of studies have been performed to assess the prevalence of HPV genotypes in several provinces in China, such as Henan, Qingdao and Yunnan^9–11^, few large samples studies have been reported.

In this study, a total of 961,029 cervical samples from women attending the gynecological outpatient were screened for HPV genotype in southeastern China (Zhejiang) between 2011 and 2015, and a subset of women also received a cytology test. We assessed the prevalence and genotype distribution of HPV in this region, and analyzed the association between cervical cytology and HPV genotype. In addition, the study will provide guidance for future vaccination programs.

## Results

### Prevalence of HPV infection in Zhejiang Province

During the 5-year study period, between January 1, 2011, and December 30, 2015, a total of 961,029 HPV tests were performed in the microbiology laboratory, Zhejiang DiAn Diagnostics. The age of the women ranged from 16 to 83 years, and the mean age was 37.34 years. The number of HPV tests increased dramatically between 2011 and 2015, and increased from 95,796 tests performed in 2011 to 281,804 in 2015, representing an almost three-fold increase. The overall HPV-positive rate during the study period was 20.54% (197,367/961,029), and the HR-HPV rate was 16.61% (159,563/961,029). The positive rate was significantly different among the different years (χ^2^ = 5516, *P* < 0.001), but a trend was not evident. The prevalence of HPV in different years is showed in Table 1. Of the positive cases, 80.85% (159,563/197,367) were HR-HPV infections, 12.40% (24,464/197,367) were LR-HPV infections, and only 6.76% (13,340/197,367) were HR & LR- HPV infections. In addition, among the HPV-positive women, 153,006 were positive for a single HPV type (153,006/197,367 = 77.52% of HPV infections; 153,006/961,029 = 15.92% of all samples), and 44,361 were positive for multiple types (44,361/197,367 = 22.48% of HPV infections; 44,361/961,029 = 4.62% of all samples). Among the 44,361 individuals infected with multiple HPV types, 34,020 had dual infections (34,020/44,361 = 76.69%), 7,493 had triple infections (7,493/44,361 = 16.89%), and 2,848 had four or more infections (2,848/44,361 = 6.42%).

**Table 1:** Prevalence of HPV in different years.

Year	Age (mean)	Total No.	HR-HPV No. (%)		LR-HPV	HR & LR- HPV	Positive case
			Single infection	Multiple infection	No. (%)	No. (%)	No. (%)
2011	36	95796	19597 (20.46%)	2537 (2.65%)	4089 (4.27%)	1289 (1.35%)	27512 (28.72%)
2012	35.72	165090	26300 (15.93%)	4747 (2.88%)	4005 (2.43%)	1382 (0.84%)	36434 (22.07%)
2013	36.71	178542	22284 (12.48%)	6109 (3.42%)	3457 (1.94%)	2276 (1.27%)	34126 (19.11%)
2014	37.54	239797	27063 (11.29%)	7423 (3.10%)	4873 (2.03%)	3353 (1.40%)	42712 (17.81%)
2015	38.98	281804	34065 (12.09%)	9438 (3.35%)	8040 (2.85%)	5040 (1.79%)	56583 (20.08%)
Total	37.34	961029	129309 (13.46%)	30254 (3.15%)	24464 (2.55%)	13340 (1.39%)	197367 (20.54%)

### Age-specific prevalence of HPV infection

The prevalence of HPV infection was significantly different among the different age groups (χ^2^ = 6514, *P* < 0.001) and peaked in the ≤20 year adolescent group (38.18%) and 61- to 70-year elderly group (26.66%). Lower HPV-positive rates were observed in the intervening age groups, with the second peak in HPV-positive rates beginning to emerge in the 51- to 60-year age group. When the ≤20 years adolescent group is excluded, the HPV-positive rate was significantly higher in women 50 years and older (23.43%, 25,176/107,432) than in women aged 21 to 50 years (19.43%, 155,128/798,415) (χ^2^ = 948.684, *P* < 0.001). The prevalence of HR-HPV infections also exhibited two similar peaks. The age-stratified HPV DNA prevalence in 10-year age groups is shown in Table 2.

**Table 2:** Prevalence of HPV in different age groups.

Age (years)	Age (mean)	Total No.	HR-HPV No. (%)		LR-HPV	HR & LR- HPV	Positive case
			Single infection	Multiple infection	(%)	(%)	(%)
≤20	18.67	11607	1827 (15.74%)	756 (6.51%)	936 (8.06%)	913 (7.87%)	4432 (38.18%)
21–30	26.52	192117	25703 (13.38%)	6639 (3.46%)	6381 (3.32%)	3653 (1.90%)	42376 (22.06%)
31–40	35.70	305567	38501 (12.60%)	8044 (2.63%)	6494 (2.13%)	2971 (0.97%)	56010 (18.33%)
41–50	45.05	300731	39535 (13.15%)	8191 (2.72%)	6166 (2.05%)	2850 (0.95%)	56742 (18.87%)
51–60	54.21	86635	12414 (14.33%)	3529 (4.07%)	2301 (2.66%)	1537 (1.77%)	19781 (22.83%)
61–70	64.01	17564	2684 (15.28%)	1007 (5.73%)	460 (2.62%)	531 (3.02%)	4682 (26.66%)
≥70	76.53	3233	405 (12.53%)	146 (4.52%)	75 (2.32%)	78 (2.41%)	704 (21.78%)
Unknown	—	43575	8240 (18.91%)	1942 (4.46%)	1651 (3.79%)	807 (1.85%)	12640 (29.01%)

### Distribution and genotypes of HPV infections

The positive rate of each genotype among all of the samples and the distribution of each genotype in the HPV-positive individuals are listed in Table 3. The most common HR-HPV types were HPV16 (17.64%), HPV52 (16.35%) and HPV58 (15.80%), and the most common LR-HPV types were HPV11 (5.61%), HPV6 (5.60%) and HPV42 (4.03%).

**Table 3:** The prevalence of each HPV genotype.

HPV Genotypes	Positive Samples	Positive Rate (in 961,029 total samples) %	Proportion (in 197,367 HPV-positive samples) %
HR-HPV			
16	34810	3.62	17.64
18	14206	1.48	7.20
31	9798	1.02	4.96
33	13149	1.37	6.66
35	6809	0.71	3.45
39	9840	1.02	4.99
45	3631	0.38	1.84
51	9999	1.04	5.07
52	32265	3.36	16.35
53	13548	1.41	6.86
56	10034	1.04	5.08
58	31182	3.24	15.80
59	5383	0.56	2.73
66	9002	0.94	4.56
68	12081	1.26	6.12
73	1588	0.17	0.80
83	876	0.09	0.44
MM4/82	2597	0.27	1.32
LR-HPV			
6	11047	1.15	5.60
11	11076	1.15	5.61
42	7960	0.83	4.03
43	3768	0.39	1.91
44	110	0.01	0.06

**Figure 1:**
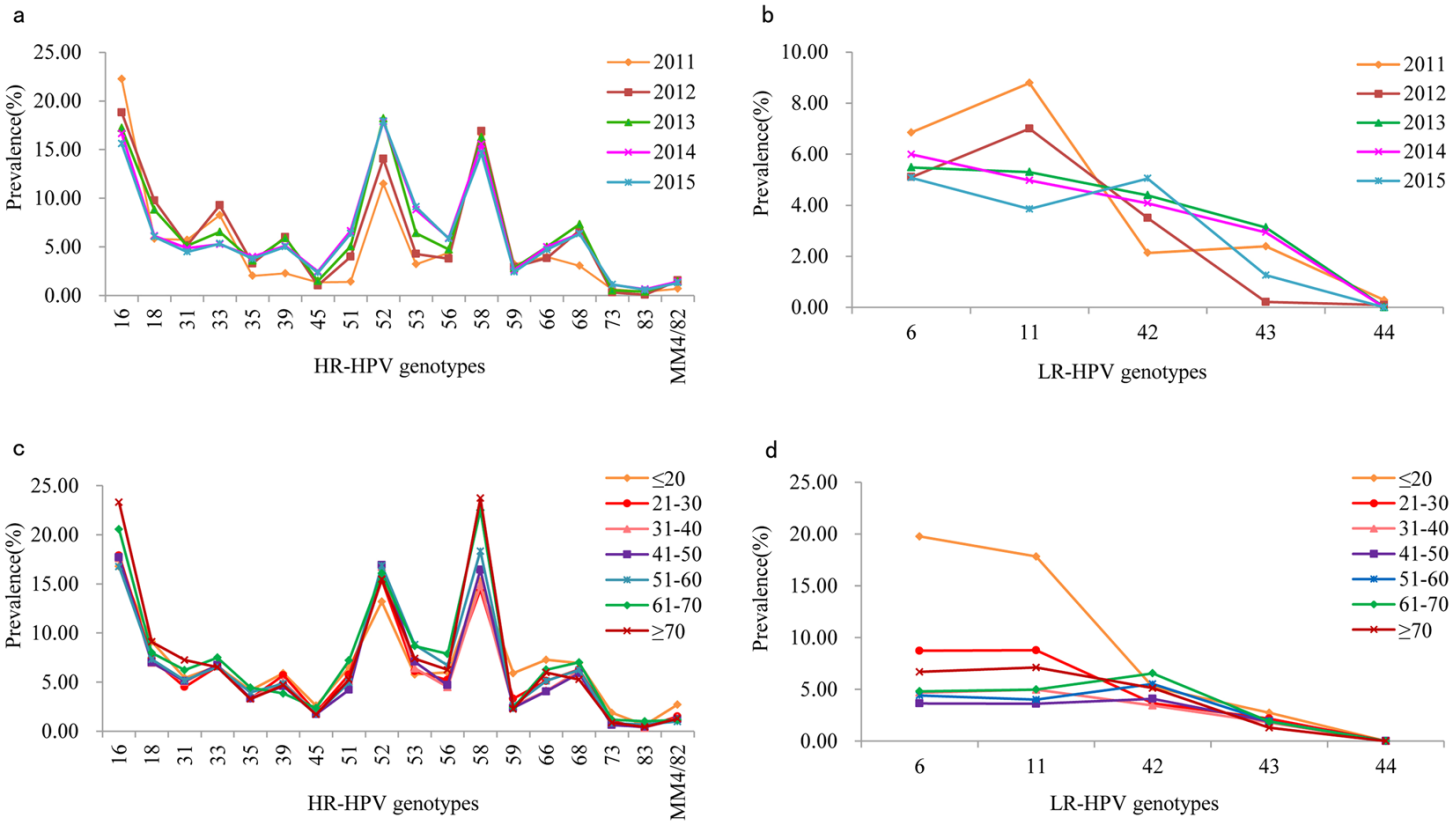
Distribution of HPV genotypes in HPV-positive women in different years and different age groups. (**a**) HR-HPV genotypes distribution. (**b**) LR-HPV genotypes distribution. (**c**) HR-HPV genotypes distribution. (**d**) LR-HPV genotypes distribution.

Of the positive cases, the HR-HPV prevalence of each type was significantly different among the different years (χ^2^ = 1705, *P* < 0.001), and the results are showed in Fig. 1a. The three most prevalent HR-HPV types in 2011–2012 were HPV16, 58 and 52, whereas the most prevalent types in 2013–2015 were HPV52, 16 and 58. The proportion of HPV16, 18, 31, 33, 39 and 59 genotypes gradually decreased. However, the HPV53 and 73 genotypes gradually increased. Between 2011 and 2015, the proportion of HPV6 fluctuated between 5% and 7%; with HPV11 decreasing dramatically from 8.79% in 2011 to 3.85% in 2015 and HPV42 increasing from 2.13% in 2011 to 5.05% in 2015. The LR-HPV distribution of each type among the different years is shown in Fig. 1b.

Among the positive cases, the HR-HPV prevalence of each type was significantly different among the different age groups (χ^2^ = 6.454, *P* = 0.011) (Fig. 1c). HPV16, 52 and 58 were always the three most prevalent HR-HPV types in each age group; however, the rank varied. HPV16 was the most prevalent in the ≤20, 21–30, 31–40, and 41–50-year-old groups. HPV58 was the most prevalent in 51–60, 61–70, ≥70- year-old groups. HPV18 was the fourth most prevalent in all age groups except for the 51–60, 61–70-year old groups (HPV 53). The LR-HPV prevalence of each type was significantly different among the different age groups (χ^2^ = 591.991, *P* < 0.001) (Fig. 1d). The most prevalent types were HPV6 in the ≤20-years group, HPV11 in 21–40-year and ≥70-year groups, and HPV42 in the 41–70-year groups.

**Figure 2:**
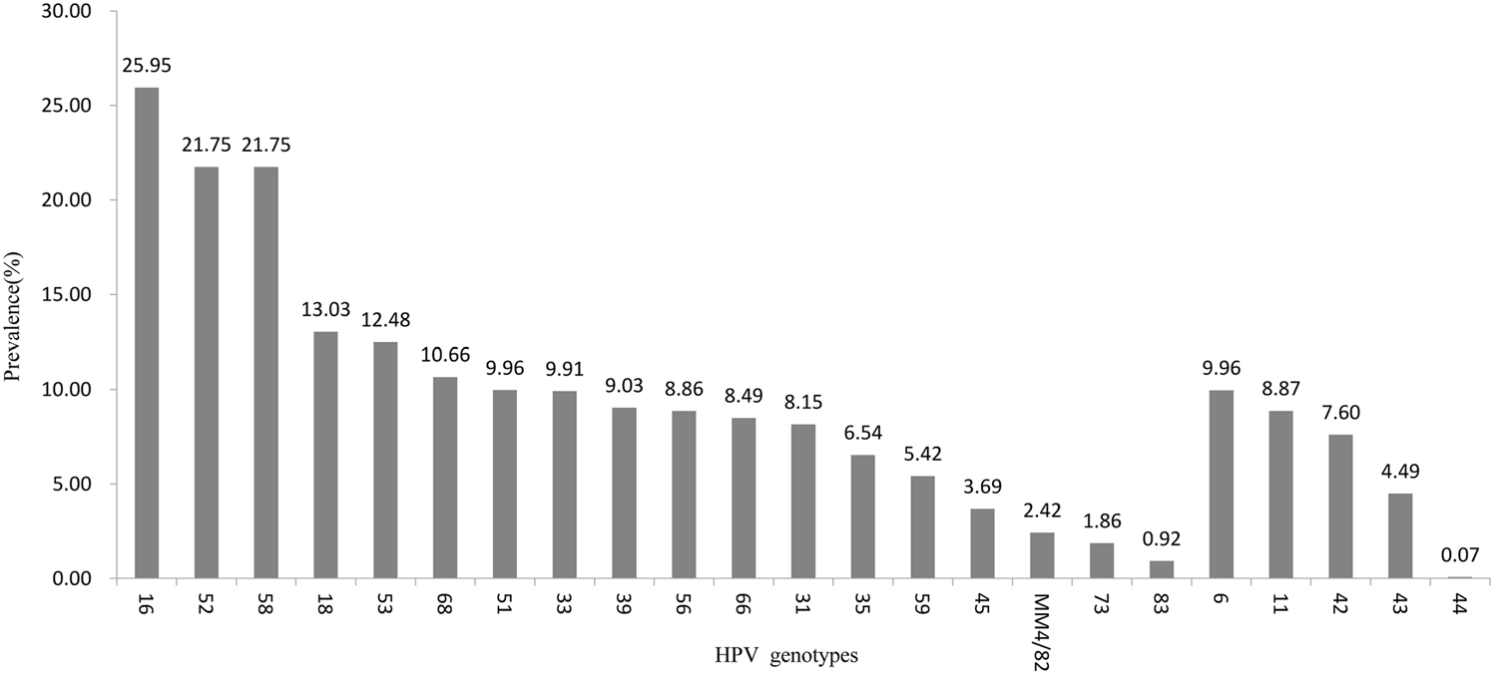
Prevalence of each HPV genotype in multiple infections.

Of the 44,361 multiple HPV positive cases, three genotypes showed higher positive rates: HPV16 (25.95%), HPV52 (21.75%), and HPV58 (21.75%) (Fig. 2). The most common combinations of genotypes were HPV16 + HPV58 (n = 1214), HPV16 + HPV18 (n = 1116), HPV16 + HPV 52 (n = 1025).

### Association between cervical cytology and HPV genotype

The association between cervical cytology and the HPV genotype of 443,890 women was analyzed. The ASC-US, LSIL and HSIL cytology accounted for a larger portion of the HR-HPV group than the LR-HPV and negative group. The abnormal cytology percentage was different among different HP-HPV type groups (χ^2^ = 2051.136, *P* < 0.001), such as 34.01% in the HPV 16 group, followed by HPV 33 (31.08%) and HPV 58 group (30.53%) (Table 4). Moreover, the HR-HPV types accounted for a larger portion in the ASC-US (64.03% vs. 7.94%), LSIL (80.04% vs. 9.70%) and LSIL (91.06% vs. 2.37%) cytology group than LR-HPV types. HPV16 and HPV58 were the major genotypes in the ASC-US, LSIL or HSIL cytology groups, followed by HPV 52 in the ASC-US and LSIL cytology groups and HPV 33 in the LSIL cytology group. HPV52, 16 and 58 were the major genotypes in the normal cytology group (Table 5).

**Table 4:** Cytology score in different HPV groups.

	No.	Normal No. (%)	ASCUS No. (%)	LSIL No. (%)	HSIL No. (%)
HR-HPV positive	86235	62638 (72.64%)	14842 (17.21%)	8062 (9.35%)	693 (0.80%)
16	13101	8646 (65.99%)	2865 (21.87%)	1238 (9.45%)	352 (2.69%)
18	5460	4016 (73.55%)	899 (16.47%)	505 (9.25%)	40 (0.73%)
31	3769	2776 (73.65%)	685 (18.17%)	273 (7.24%)	35 (0.93%)
33	5070	3494 (68.92%)	966 (19.05%)	528 (10.41%)	82 (1.62%)
35	2736	2085 (76.21%)	423 (15.46%)	216 (7.89%)	12 (0.44%)
39	3892	2995 (76.95%)	611 (15.70%)	278 (7.14%)	8 (0.21%)
45	1451	1172 (80.77%)	209 (14.40%)	67 (4.62%)	3 (0.21%)
51	3998	2888 (72.24%)	655 (16.38%)	449 (11.23%)	6 (0.15%)
52	13010	9832 (75.57%)	2202 (16.93%)	943 (7.25%)	33 (0.25%)
53	5528	4200 (75.98%)	734 (13.28%)	586 (10.60%)	8 (0.14%)
56	4034	2896 (71.79%)	542 (13.44%)	593 (14.70%)	3 (0.07%)
58	11737	8153 (69.46%)	2292 (19.53%)	1200 (10.22%)	92 (0.78%)
59	2085	1568 (75.20%)	362 (17.36%)	153 (7.34%)	2 (0.10%)
66	3418	2404 (70.33%)	433 (12.67%)	578 (16.91%)	3 (0.09%)
68	4873	3885 (79.73%)	666 (13.67%)	320 (6.57%)	2 (0.04%)
73	661	528 (79.88%)	92 (13.92%)	39 (5.90%)	2 (0.30%)
MM4/82	1079	821 (76.09%)	173 (16.03%)	75 (6.95%)	10 (0.93%)
83	333	279 (83.78%)	33 (9.91%)	21 (6.31%)	0 (0.00%)
LR-HPV positive	14117	11281 (79.91%)	1841 (13.04%)	977 (6.92%)	18 (0.13%)
HPV negative	343538	335958 (97.79%)	6497 (1.89%)	1033 (0.30%)	50 (0.01%)
Total	443890	409877(92.34%)	23180 (5.22%)	10072 (2.27%)	761 (0.17%)

**Table 5:** HPV genotype distribution in different cytological results.

HPV type	Normal (409877) No. (%)	ASCUS (23180) No. (%)	LSIL (10072) No. (%)	HSIL (761) No. (%)
HR-HPV positive	62638 (15.28%)	14842 (64.03%)	8062 (80.04%)	693 (91.06%)
16	8646 (2.11%)	2865 (12.36%)	1238 (12.29%)	352 (46.25%)
18	4016 (0.98%)	899 (3.88%)	505 (5.01%)	40 (5.26%)
31	2776 (0.68%)	685 (2.96%)	273 (2.71%)	35 (4.6%)
33	3494 (0.85%)	966 (4.17%)	528 (5.24%)	82 (10.78%)
35	2085 (0.51%)	423 (1.82%)	216 (2.14%)	12 (1.58%)
39	2995 (0.73%)	611 (2.64%)	278 (2.76%)	8 (1.05%)
45	1172 (0.29%)	209 (0.9%)	67 (0.67%)	3 (0.39%)
51	2888 (0.7%)	655 (2.83%)	449 (4.46%)	6 (0.79%)
52	9832 (2.4%)	2202 (9.5%)	943 (9.36%)	33 (4.34%)
53	4200 (1.02%)	734 (3.17%)	586 (5.82%)	8 (1.05%)
56	2896 (0.71%)	542 (2.34%)	593 (5.89%)	3 (0.39%)
58	8153 (1.99%)	2292 (9.89%)	1200 (11.91%)	92 (12.09%)
59	1568 (0.38%)	362 (1.56%)	153 (1.52%)	2 (0.26%)
66	2404 (0.59%)	433 (1.87%)	578 (5.74%)	3 (0.39%)
68	3885 (0.95%)	666 (2.87%)	320 (3.18%)	2 (0.26%)
73	528 (0.13%)	92 (0.4%)	39 (0.39%)	2 (0.26%)
MM4/82	821 (0.2%)	173 (0.75%)	75 (0.74%)	10 (1.31%)
83	279 (0.07%)	33 (0.14%)	21 (0.21%)	0 (0%)
LR-HPV positive	11281 (2.75%)	1841 (7.94%)	977 (9.70%)	18 (2.37%)
HPV negative	335958 (81.97%)	6497 (28.03%)	1033 (10.26%)	50 (6.57%)

**Figure 3:**
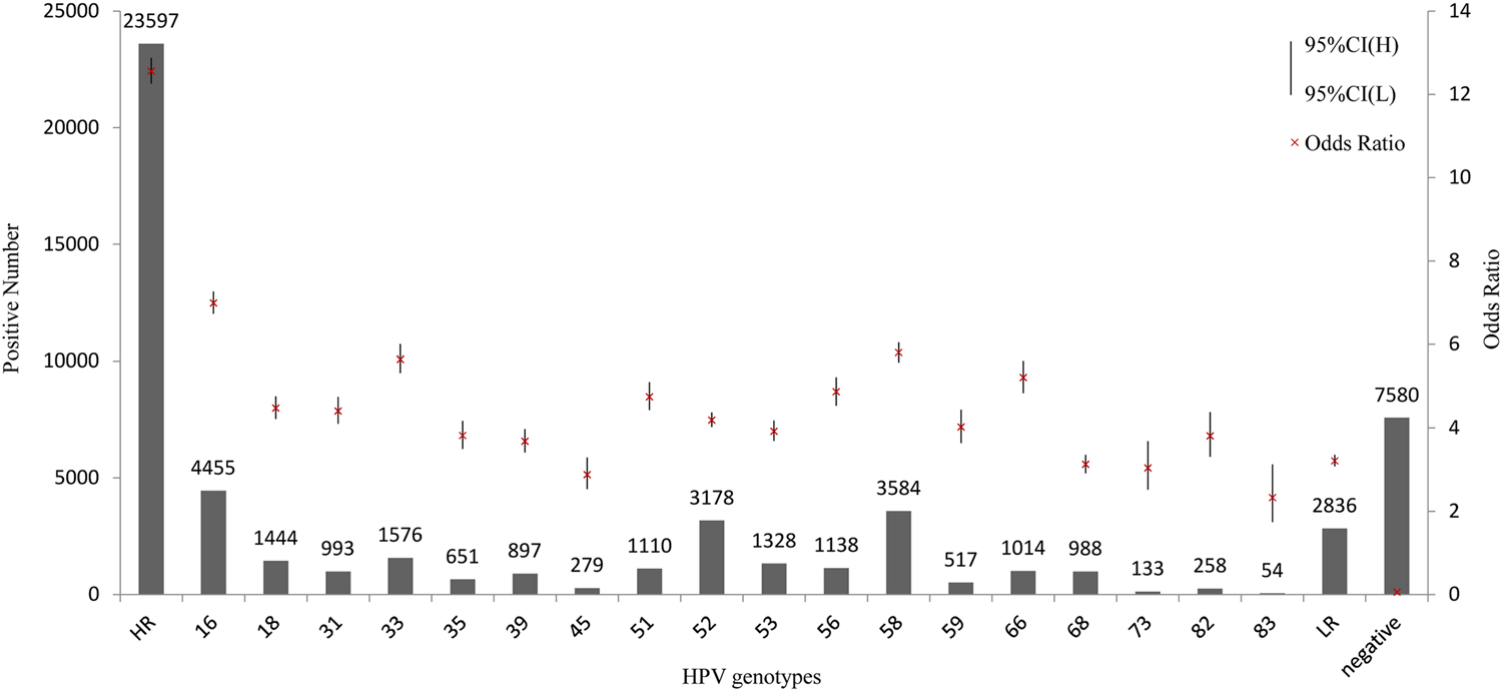
Risk of each HPV type for cervical lesions. Crude odds ratios with 95% confidence intervals for the prevalence of HPV associated with abnormal cytology (ASC-US, LSIL, HSIL) are provide.

To estimate the risk of HPV infection with abnormal cytology, the crude ORs for the prevalence of HPV associated with abnormal cytology (ASC-US, LSIL and HSIL) were determined and are shown in Fig. 3. The ORs of the HR-HPV and LR-HPV infection and HPV-negative groups were 12.56, 3.21 and 0.06, respectively. Among genotypes, HPV 16 had the highest OR (6.99), followed by HPV 58 (5.80), 33 (5.65), and 66 (5.21).

## Discussion

This is one of the few investigations of HR and LR-HPV in a large-scale screen of the population of southeastern China (Zhejiang Province). More than 960,000 samples were collected from 2021 widely distributed institutions in large cities, urban areas and rural areas of Zhejiang Province. The study population consisted of gynecological outpatients and asymptomatic women.

In this present survey, the overall HPV-positive rate was 20.54%, a rate similar to that in a previous cross-sectional study in Zhejiang (22.8%)^12^, higher than that in Yunnan (12.9%)^11^, and lower that in Henan (44.5%)^9^ and Qingdao (32.2%)^10^. In this study, a downward trend in the HPV-positive rate was evident from 2011 to 2014 (from 28.72% in 2011 to 17.81% in 2014). Similarly, the HR-HPV-positive rate was found to decline from 25.3% in 2007 to 18.4% in 2014 in Guangzhou^13^. The decline in prevalence may be caused by the following reasons: (1) With the increasing attention to cervical cancer, many healthy people (asymptomatic population) attended HPV screening, which results in the prevalence of HPV tending to be natural infection rate. The HPV prevalence in women without cervical abnormalities varied from 1.6% to 14.2% in Asia countries^14^, and 13.3% in Zhejiang^15^. (2) Some HPV-positive women underwent HPV detection in the following years, and the result might turn out to be negative due to the immune clearance and treatment. Interestingly, the prevalence of HPV increased to 20.08% in 2015 (higher than 2013 and 2014), which may be attributed to the change of cervical cancer screening method. HPV detection has been the primary method for cervical cancer screening in clinics since 2010, while cytology test has been emphasized due to it’s higher specificity since nearly 2015. Some women showed abnormal cervical cytology, were then carried out HPV detection, which contributed to the higher prevalence of HPV. However, a nationwide investigation in China showed that the HR-HPV infection rate is increasing in many regions, such as Haikou, Chongqing, and several cities in the north^16^. The reported prevalence varies among studies because of regional differences, the population composition, the sampling periods and the testing methods.

The general age distribution showed an initial HPV peak in the ≤20-years group, a decreasing tread in the middle-age group, and a significantly increasing tread in the >50-years group. This bimodal pattern has been observed in several other studies in China^9–13^ and may have been caused immunosenescence, changes in sexual behavior during middle age, persistent viral infection, or reactivation.

Consistently with the data generated by Chinese population-specific investigations, HPV16, HPV52, and HPV58 were found to be the dominant HR-HPV types^10,16,17^. In contrast to the data reported by Wang *et al*.^16^, who have performed a nationwide population-based investigation in 37 cities in China, the infection rates of HPV16 (4.82%) and HPV52 (4.52%) were higher than the rates of HPV16 (3.62%) and HPV52 (3.36%) in the present study, whereas the rate of HPV58 was lower (2.74% vs 3.24%). In addition to HPV16, HPV18 is also important for cervical carcinogenesis^5^. In our present study, it was the fourth most common infection, and the infection rate (1.48%) was consistent with the results of studies by Liu *et al*.^12^ and Wang *et al*.^16^. Many benign cutaneous warts, mucosal lesions, and low-grade cervical intraepithelial lesions generate a considerable health burden associated with LR-HPV infection^18^. In our present survey, the incidence of HPV6 (1.15%) and HPV11 (1.15%) infection was higher than that in Asia in a 5-continent study (HPV6 0.2%, HPV11 0.1%)^19^, and lower than that in nationwide investigation (HPV6 4.01%, HPV11 2.29%)^16^.

From 2011 to 2015, HPV 16, 52, and 58 were always the major types in each age group and year. However, the ranks varied with the year and age. HPV16 was the most prevalent in the ≤50-years group, whereas HPV58 was the most prevalent in the >50-years group. The change may have been related to physiological and immunological disorders associated with hormone fluctuations. Moreover, between 2011 and 2015, the prevalence of the HPV53 and 73 genotypes gradually increased, whereas the HPV16, 18, 31, 33, 39 and 59 genotypes decreased. Although the reason for these variations is unclear, this phenomenon should be monitored via surveys.

To date, several studies have shown that multiple HPV infections influence the duration of type-specific episodes and cervical cancer development. For example, Lee *et al*.^20^ have reported an association between infection with multiple HPV types and an increased risk of cervical cancer. Additionally, Schmitt *et al*.^21^ have confirmed that co-infection increases the duration of infection. Furthermore, patients with multiple high viral loads show a 4- to 6-fold increased risk of cervical precancerous cytological lesions compared with the risk in patients with single high viral loads. Recently, Adela *et al*.^22^ have reported that compared with infection with either HPV16 or 68 alone, coinfection with HPV68 and 16 increases the risks of HSIL and ICC. Thus, the characterization of the prevalence of multiple HPV infections might be important for understanding its effects on cervical carcinogenesis and the prognosis of patients with persistent infection. In the present study, the proportion of individuals with multiple infections among the HPV-positive women was 22.48%, a value lower than those reported in Shanghai (36.6%) and Beijing (27.7%) and similar to those reported in Shanxi (24.3%)^23^. The most common combinations of genotypes were HPV16 + HPV58, HPV16 + HPV18 and HPV16 + HPV 52, a result inconsistent with the results of Wang *et al*.^16^, who have reported that HPV16 + HPV52 and HPV52 + HPV58 were the most frequent.

In this study, the HPV prevalence in the normal, ASC-US, LSIL and HSIL cytology samples was 18.03%, 71.97%, 89.74% and 93.43%, respectively. The same trend was also observed in other regions or countries. A population-based study of HPV genotype prevalence in America has reported that the overall HPV infections were found in 24.3%, 57.9%, 94.6% and 95.5% of the normal, ASC-US, LSIL and HSIL cytology samples, respectively^24^. In Henan, central China, the overall HPV prevalence in the normal, ASC-US, LSIL and HSIL cytology samples have reported to be 57.1%, 72.5%, 84.0% and 88.6%, respectively^25^. Additionally, in this study, the ORs for the prevalence of the HR-HPV and LR-HPV infections and HPV-negative groups with the abnormal cytology were 12.56, 3.21 and 0.06, respectively. These results indicate that the association between HPV infection and cervical neoplasia is strong. HPV16, 52, 58 and 18 have been found to be the dominant HR-HPV types in this area, and HPV 16 has been found to have the highest OR (6.99) for abnormal cytology, followed by HPV58 (5.80), 18 (4.48), 52 (4.19). These findings indicated that in addition to HPV16 and HPV18, the HPV vaccine in China might also include the HPV52 and HPV58 genotypes. Notably, the proportion of HPV53 and 73 genotypes in this study gradually increased between 2011 and 2015, and the ORs of the genotypes were 3.92 and 3.04, respectively. However, certain studies have shown that HPV 73 is even more risky than HPV 16, on the base of HPV-type-specific risk analysis in Bahia, Brazil^26^ and northwest Germany^27^. Further studies on HPV 73 should be performed.

In conclusion, the most significant findings of this survey of more than 960,000 samples in Zhejiang Province are as follows: (1) the prevalence of HPV infection showed population variations in age and years; (2) HPV 16, 52, and 58 were always the major genotypes, although the rank varied according to the year and age; and (3) HPV prevalence increased as the lesions progressed to higher grades. HPV 16, 58, 33, 66 were the most risky types in this area. Our results will provide guidance for primary screening and vaccination program for cervical cancer.

## Materials and Methods

DiAn Diagnostics is the largest independent laboratory in China, and it is certified by the International Standardization Organization (ISO15189) and College of American Pathologists (CAP). HPV genotype and ThinPrep cytology test (TCT) are covered under the checklist of the accreditation program. This investigation involved 2021 clinical hospitals, women’s health centers, and clinics located in 11 different regions of Zhejiang Province between 2011 and 2015.

### Ethical statement

This study was approved by the Ethical Review Board of the Zhejiang Provincial People’s Hospital. All methods were carried out in accordance with the approved guidelines and the Declaration of Helsinki. Written informed consent was obtained from each study participant.

### Cervical specimen collection

Cervical exfoliated cells from women attending the gynecological outpatient were collected using specialized cervical brush by clinician. The cervical samples were kept in sample transport medium for HPV detection and cell preservation solution for cytology test, respectively. All samples were shipped to the lab at 4 °C and performed the test within 48 hours.

### HPV Genotyping

HPV detection and genotyping were performed using a commercial HPV Genotyping Kit for 23 HPV tests (Yaneng Biotechnology Limited Corp. Shenzhen, China), including 18 HR-HPV types (16, 18, 31, 33, 35, 39, 45, 51, 52, 53, 56, 58, 59, 66, 68, 73, 82 and 83) and 5 LR-HPV types (6, 11, 42, 43 and 44). The kit employs DNA amplification to detect HPV positivity and a microarray format with a nylon membrane onto which HPV genotype-specific oligonucleotide probes have been immobilized to simultaneously identify 23 HPV genotypes. The technique was validated through the use of positive and negative controls at each shift. This kit has been approved by the China Food and Drug Administration (CFDA) and Conformite Europeenne (CE). The experimental procedure was performed according to the manufacturer’s instructions, and the protocol had been described in a previous study^10^, including DNA extraction, polymerase chain reaction (PCR) and reverse dot blot hybridization (RDB). The supernatants were removed by centrifugation at 13000 rpm for 10 min. The cell deposit well was mixed 50 μl lysates, and incubated in 100 °C for 10 min, then centrifuged at 13000 rpm for 10 min. DNA was in the supernatant. The PCR was carried out in 25 μl reaction mixture in a thermal cycler and the cycling parameters were as follows: 50 °C for 15 min, 95 °C for 10 min, followed by 40 cycles (30 s at 94 °C, 90 s at 42 °C and 30 s at 72 °C), with a final extension at 72 °C for 5 min. After amplification, HPV genotyping was done by hybridization and RDB on the strips fixed with 23 different type-specific probes. The blue spots on the strip could be judged as positive by the naked eye.

### ThinPrep cytology test and pathological diagnosis

The Cervical exfoliated cells were made into smear with a diameter of 2 cm, immobilized with 95% alcohol and stained by Pap staining. Cytological slides were read by experienced pathologists. Cytological cell samples were categorized according to the Bethesda system criteria (2001)^28^ as follows: negative for intraepithelial lesion or malignancy (NILM), atypical squamous cells of undetermined significance (ASC-US), low-grade squamous intraepithelial lesions (LSIL), and high-grade squamous intraepithelial lesions (HSIL).

### Statistical Analysis

Statistical analyses were performed using SPSS version 17.0 for Windows. The chi-square test was used to compare the prevalence or proportions between different groups, with crude odds ratios (ORs) and a 95% confidence intervals (CIs) calculated to estimate the risk of each HPV type for cervical lesions. *P*-values were two-sided, and statistical significance was defined as *P* < 0.05.
